# Study on Nutritional Characteristics, Antioxidant Activity, and Volatile Compounds in *Non*-*Saccharomyces cerevisiae*–*Lactiplantibacillus plantarum* Co-Fermented Prune Juice

**DOI:** 10.3390/foods14111966

**Published:** 2025-05-31

**Authors:** Yu Zhao, Rui Yang, Wei Wang, Tongle Sun, Xinyao Han, Mingxun Ai, Shihao Huang

**Affiliations:** College of Food Science and Pharmacy, Xinjiang Agricultural University, Urumqi 830052, China; zy99370891@163.com (Y.Z.); yrui1220@163.com (R.Y.); ms-stl@outlook.com (T.S.); 18340383068@163.com (X.H.); 15662319262@163.com (M.A.); s08111119cau@163.com (S.H.)

**Keywords:** fermented prune juice, *Non-Saccharomyces cerevisiae*, *Lactiplantibacillus plantarum*, physicochemical properties, antioxidant capacity, volatile compounds

## Abstract

The fermentation of prune juice significantly enhances its nutritional profile, antioxidant capacity, and flavor characteristics. In this study, *Non-Saccharomyces cerevisiae* and *Lactiplantibacillus plantarum* were used to co-ferment prune juice to systematically investigate the dynamic changes in physicochemical properties and antioxidant activity during fermentation. The evolution of volatile compounds across fermentation stages was analyzed using gas chromatography–ion mobility spectroscopy (GC-IMS) combined with chemometric methods, including principal component analysis (PCA) and orthogonal partial least squares discriminant analysis (OPLS-DA). The results showed that after fermentation, the total acidity (TA), total phenolic content (TPC), and total flavonoid content (TFC) increased by 37.35%, 20.28%, and 28.95%, respectively. Meanwhile, the pH, total soluble solids (TSS), and reducing sugars (RS) decreased by 16.87%, 23.36%, and 39.94%, respectively. Additionally, the DPPH radical scavenging capacity and ABTS radical scavenging capacity improved by 76.16% and 57.25% during fermentation process. A total of 37 volatile compounds were identified across the four fermentation stages of prune juice (PJ). These compounds included 14 esters, 8 alcohols, 7 aldehydes, 4 terpenoids, 3 ketones, and 1 amine. Considerable quantities of organic acids and free amino acids were detected in samples from all fermentation phases. Among these, lactic acid, citric acid, and D-glucuronic acid exhibited significant increases in their concentration (*p* < 0.05). In the free amino acid profile of fermented prune juice (FPJ), asparagine was the most abundant component, followed by glutamine and proline.

## 1. Introduction

The prune, originating from the European plum (*Prunus domestica* L.) [1], is a highly nutrient-dense fruit abundant in anthocyanins, phenolic compounds, flavonoids, dietary fiber, and essential trace minerals. These bioactive components offer substantial physiological advantages, such as antioxidant properties, immune system fortification, and cardiovascular protection [2]. In Kashgar Prefecture, Xinjiang, prune production reached 350,000 metric tons in 2024 [3]. The development of advanced deep-processing technologies for prunes has significantly mitigated storage and distribution challenges arising from the high postharvest respiratory rate and inherent susceptibility of fresh prune fruits to softening and decay. Currently, the value-added processing of prunes predominantly centers on products like dried prunes, candied fruits, and jams. However, investigations into fermented prune juice remain conspicuously scarce. Fermented fruit juice is a functional beverage synthesized via the biotransformation of fresh fruits by probiotic microorganisms (such as lactic acid bacteria, yeast, or acetic acid bacteria) under precisely controlled conditions. This fermentation process yields a diverse array of bioactive compounds, encompassing minerals, amino acids, total phenolic compounds, organic acids, and polysaccharides. These compounds collectively endow the beverage with multiple health-promoting properties, including free radical scavenging, anti-inflammatory activity, immune system modulation, the regulation of glycolipid metabolism, anti-fatigue effects, and antimicrobial capabilities [4].

Probiotic fermentation of fresh fruits has the potential to modify the composition of flavor components and bioactive substances within the raw materials [5]. Specifically, it can lead to changes in the types and concentrations of these components. Studies have shown that lactic acid fermentation, particularly in the context of lactic acid-fermented prune juice, significantly boosts the TPC and TFC in fermented mulberry juice. This increase in TPC and TFC contributes to an enhancement of the juice’s antioxidant activity [6]. Liu et al. [7] carried out a study where they used coupled fermentation with lactic acid bacteria (LAB) and yeast in jujube juice. Through this approach, they identified 53 volatile compounds. Their research further demonstrated that mixed-culture fermentation effectively improves the sensory quality, such as flavor and aroma, as well as the nutritional quality, like the content of beneficial compounds, of fermented jujube juice. Mandha et al. [8] identified 44 volatile compounds in fermented mango juice, noting significant increases in ketones (e.g., 3-hydroxy-2-butanone) and furans (e.g., furfural), which endow the fermented mango juice with fruity, sweet, and toasted aromas. Salas-Millàn and Aguayo [9] reported that lactic acid bacteria-fermented broccoli–radish beverages contained 16 amino acids, including 8 essential amino acids (e.g., leucine and lysine) and 8 non-essential amino acids (e.g., glutamic acid and proline). Bi et al. [10] utilized high-performance liquid chromatography (HPLC) to investigate the dynamic changes in organic acids during pear juice fermentation, revealing a biphasic trend in total organic acid content: an initial decrease during early fermentation followed by a subsequent increase with prolonged fermentation. While previous studies have preliminarily explored the functional and nutritional attributes of fermented fruit juices, systematic investigations into fermented prune juice—specifically the stage-dependent evolution of its physicochemical properties, antioxidant capacity, and volatile compound profiles—remain largely unreported in the current literature. The development of FPJ not only expands the utilization of prune resources and boosts economic benefits in the prune industry but also offers consumers a novel beverage that integrates nutritional and health benefits. This aligns with the modern food industry’s requirements for functionality, naturalness, and sustainability.

This study takes fermented prune juice as the experimental material. It systematically investigates the physicochemical parameters, antioxidant capacity, and volatile compound profiles of the juice during various fermentation stages. By integrating advanced analytical techniques, including gas chromatography–ion mobility spectrometry (GC-IMS), principal component analysis (PCA), and orthogonal partial least squares discriminant analysis (OPLS-DA), we aim to unravel the dynamic evolution of volatile compounds and uncover the relationships between fermentation phases and functional/nutritional attributes.

## 2. Materials and Methods

### 2.1. Materials

The prune samples were harvested from Kashgar, Xinjiang. *Lactiplantibacillus plantarum* was obtained from Chr. Hansen A/S, a renowned biotechnology company in Denmark, and stored at −45 °C. *Non-Saccharomyces cerevisiae* (*Torulaspora delbrueckii*) was provided by Angel Yeast Co., Ltd. (Yichang, China) a leading yeast manufacturer in China, and stored at 4 °C. Pectinase BXL, with an enzymatic activity of 50,000 U/g, was sourced from LAFFORT, a French company specializing in oenological products. Potassium metabisulfite (E224) was acquired from OENOFRANCE, a French supplier of winemaking additives. Folin–Ciocalteu reagent, sodium hydroxide, and 2,2-diphenyl-1-picrylhydrazyl solution were purchased from Sangon Biotech Co., Ltd., a well-known provider of biochemical products in Shanghai, China. Ketones such as 2-butanone, 2-octanone, and 2-nonanone were procured from Aladdin Inc., a company specializing in chemical reagents in Shanghai, China. All solvents used in this study were of analytical grade.

### 2.2. Sample Preparation

We selected fresh prunes free of decay or spoilage. After washing and pitting, we added purified water at a solid-to-liquid ratio of 3:1 (*w*/*w*). Prune juice (soluble solids content: 20%, pH 4.0) was prepared via mechanical cell wall disruption and homogenization using a high-speed blender integrated with wall-breaking technology (SPJ1002S). Yeast and *Lactiplantibacillus plantarum* were activated in drinking water at 23 °C. We added 0.04% (*w*/*v*) composite enzyme preparation to the juice and incubated it at 44 °C for 3 h. Following enzymatic treatment, potassium metabisulfite was added to adjust the SO_2_ concentration to 60 mg/L. The juice was transferred to a fermentation tank. Fermentation was performed via sequential inoculation of yeast followed by *Lactiplantibacillus plantarum*. Firstly, we inoculated it with 0.1% activated yeast and fermented it at 28 °C for 24 h under anaerobic conditions. Subsequently, we adjusted the temperature to 37 °C, inoculated it with 0.06% activated *Lactiplantibacillus plantarum*, and continued lactic acid fermentation for 24 h under anaerobic conditions. The total fermentation duration was 48 h. The product was pasteurized at 75 °C for 15 s, clarified, and filtered using a plate-and-frame filter (CXSQW200), after which the fermented prune juice was collected. During the fermentation process, the samples were labeled as prune juice (PJ), yeast-fermented prune juice (YFPJ), lactic acid bacteria-fermented prune juice (LFPJ), and fermented prune juice (FPJ) and then cryopreserved for subsequent analysis.

### 2.3. Physicochemical Properties Analysis

The TA was determined via titration with 0.1 mol/L aqueous sodium hydroxide (NaOH), as described by Minaar et al. [11]. pH values were measured using a digital pH meter (PHB-5), and the TSS was quantified with a handheld refractometer (BM05). RS were determined using the 3,5-dinitrosalicylic acid (DNS) method, as described by Aihaiti et al. [12].

### 2.4. Determination of TPC and TFC Contents

The TPC was analyzed according to the method described by Liu et al. [13]. Absorbance was measured at 765 nm using a UV spectrophotometer, with the results calculated via the gallic acid standard curve regression equation ($y=0.0205x+0.0781,R^{2}=0.9975$) and expressed as milligrams per liter (mg/L).

For TFC, the method reported by Jikang et al. [14] was adopted. Absorbance at 510 nm was measured using a UV spectrophotometer, and samples were quantified using the rutin standard curve ($y=0.0717x+0.0537,R^{2}=0.9991$), with the results reported as mg/L.

### 2.5. Determination of Antioxidant Capacity

#### 2.5.1. DPPH Radical Scavenging Assay

The DPPH assay was performed according to the method of Wu et al. [15], with slight modifications. Briefly, 60 μL of fermented broth (1:10 dilution) was mixed with 2.1 mL of methanol-diluted DPPH solution (0.1 mM). The mixture was incubated in the dark at room temperature for 30 min, and the absorbance (*A*) was measured at 517 nm. A control group containing fermented broth without DPPH was prepared. The DPPH radical scavenging rate was calculated using the following formula:

(1) $$Scavenging\;Rate\left(\%\right)=\left(1-\frac{A_{s}}{A_{c}}\right)\times 100$$

where $A_{c}$ and $A_{s}$ correspond to the absorbance of the control and sample groups, respectively.

#### 2.5.2. ABTS Radical Scavenging Assay

The ABTS assay was adapted from that of Yuan et al. [16]. A stock solution was prepared by mixing 5 mL of ABTS solution (0.1 mM) with 90 mL of ammonium persulfate [(NH_4_) _2_S_2_O_8_, 72 mM], followed by 16 h of dark incubation at room temperature. The mixture was diluted with ethanol to achieve an absorbance of 0.70 ± 0.02 at 734 nm. Subsequently, 60 μL of the sample (1:10 dilution) was added to 2.1 mL of the diluted ABTS solution and incubated in the dark for 10 min. Absorbance (*A*) was measured at 734 nm, with a control group containing the sample without ABTS. The ABTS radical scavenging rate was calculated as:

(2) $$Scavenging\;Rate\left(\%\right)=\left(1-\frac{A_{s}}{A_{c}}\right)\times 100$$

where $A_{c}$ and $A_{s}$ correspond to the absorbance of the control and sample groups, respectively.

### 2.6. Determination of Volatile Compounds

Following the method described by Li et al. [17], fermented prune juice samples from four fermentation stages were analyzed using a Flavour Spec^®^ GC-IMS instrument (G.A.S. GmbH, Frankfurt am Main, Germany). Each sample (2.0 g) was placed into 20 mL headspace vials, with three biological replicates per sample. After labeling, the vials were loaded into the autosampler tray and equilibrated at 50 °C for 15 min. Subsequently, 500 μL of the headspace gas was automatically injected into the GC-IMS system using a heated syringe (80 °C), with a stirring speed of 500 rpm and an injector temperature of 85 °C. Semi-quantitative analysis was conducted using normalized peak areas, wherein the relative abundance of each volatile compound was calculated as the ratio of its chromatographic peak area to the total peak area of all detected volatile components [18].

### 2.7. Determination of Organic Acids

The organic acid composition and content in FPJ at different fermentation stages were analyzed using liquid chromatography–mass spectrometry (LC-MS) with a method adapted from that of Fiori et al. [19]. Chromatographic separation was performed on an ACQUITY UPLC^®^ BEH C18 column (2.1 × 100 mm, 1.7 μm) using a two-component mobile phase: (A) 50% (*v*/*v*) methanol–water (containing 0.1% formic acid) and (B) 10% (*v*/*v*) methanol–water (containing 0.1% formic acid). The flow rate was maintained at 0.4 mL/min, with an injection volume of 5 μL and a column temperature set at 40 °C. Prior to analysis, samples were diluted 1000-fold [20], vortexed for 30 s, and filtered through a 0.22 μm membrane. Qualitative identification of organic acids in FPJ was achieved by matching the retention times with those of standard compounds, while quantitative analysis was conducted using calibration curves prepared from organic acid standard solutions. Concentrations were expressed in micrograms per milliliter (μg/mL).

### 2.8. Determination of Amino Acids

The amino acid composition and content in FPJ at different fermentation stages were analyzed using LC-MS, based on a modified method reported by D. Virág et al. [21]. Chromatographic separation was performed on an ACQUITY UPLC^®^ BEH C18 column (2.1 × 100 mm, 1.7 μm) with a binary mobile phase: (A) 50% methanol in water (containing 0.1% formic acid) and (B) 10% methanol in water (containing 0.1% formic acid). A gradient elution program was applied with flow rates of 0.3 mL/min from 0 to 8.0 min and 0.4 mL/min from 8.0 to 17.5 min. The injection volume was set at 5 μL, and the column temperature was maintained at 40 °C. Prior to analysis, the supernatants were filtered through a 0.22 μm membrane [22]. Qualitative and quantitative analysis of amino acids in FPJ was conducted by comparing the retention times and mass spectral data with those of amino acid standards, while quantitative determination was performed using calibration curves generated from standard solutions, with concentrations expressed in μg/mL.

### 2.9. Statistical Analysis

All experiments were performed in triplicate, with the results presented as the mean ± standard deviation (SD). Analysis of variance (ANOVA) was performed using SPSS 27. Data analysis and graphical visualization were conducted using Microsoft Excel 2021 and Origin 2021, while multivariate statistical analyses—including principal component analysis (PCA) and OPLS-DA—were performed with SIMCA 14.1. A significance threshold of *p* < 0.05 was applied to identify statistically significant differences.

## 3. Results and Discussion

### 3.1. Changes in TA and pH of FPJ During Different Fermentation Stages

**Figure 1:**
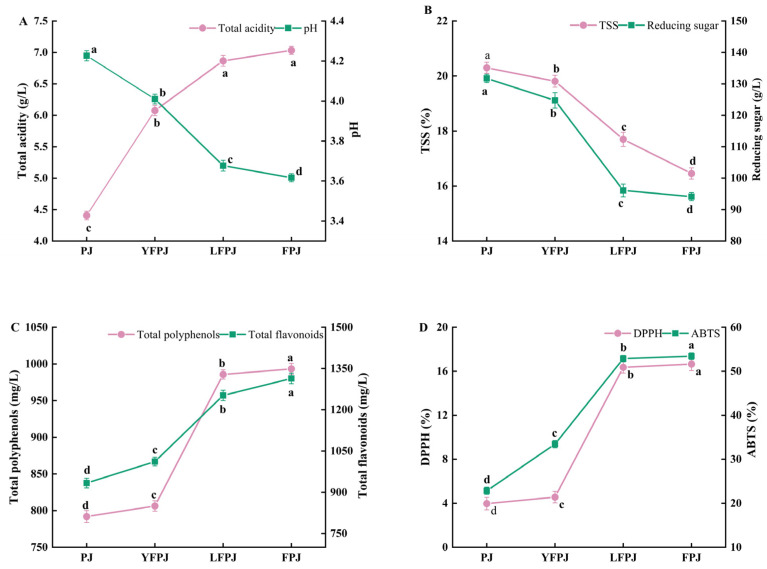
Changes in physicochemical properties (**A**,**B**), TPC and TFC (**C**), and DPPH and ABTS (**D**) at different fermentation stages. In each figure, different letters mean significant differences (*p* < 0.05).

A decrease in the pH effectively inhibits the proliferation of spoilage microorganisms, thereby prolonging the microbial shelf life [23]. As depicted in Figure 1A, the pH values of YFPJ, LFPJ, and FPJ were significantly lower (*p* < 0.05) than that of PJ, while their TA increased markedly (*p* < 0.05). Specifically, PJ exhibited a pH of 4.23 ± 0.08, whereas YFPJ, LFPJ, and FPJ displayed pH values of 4.01 ± 0.11, 3.68 ± 0.17, and 3.62 ± 0.22, respectively, representing decreases of 5.2%, 13.0%, and 14.4% relative to PJ. This pH reduction during fermentation is primarily attributed to the microbial production of organic acids (e.g., lactic acid and acetic acid), a phenomenon consistent with the findings of Sharma et al. [24]. Concurrently, total acidity showed a significant upward (*p* < 0.05) trend throughout the fermentation period, reflecting the accumulation of organic acids generated via microbial metabolism. The results indicated a rapid acidogenesis phase within the first 24 h of fermentation. By the end of the fermentation process, the TA of FPJ increased from 4.41 ± 0.04 g/L to 7.03 ± 0.54 g/L—a 37.35% increase—consistent with TA elevations reported in fermented pomegranate juice, litchi wine, and orange juice [25,26]. This TA augmentation is primarily attributed to the microbial biosynthesis of organic acids (e.g., α-ketoglutaric acid and succinic acid) via the glycerol–pyruvate fermentation pathway during alcoholic fermentation, a metabolic route well documented in fruit and vegetable juice bioconversion [27]. The concurrent decrease in pH and increase in TA align with findings from Chen et al. [28] on apple juice fermentation systems and Sun et al. [29] on fermented apricot juice, where acid production by microbial consortia was identified as the key driver of pH modulation and total acidity enhancement.

### 3.2. Changes in TSS and RS of FPJ During Different Fermentation Stages

As illustrated in Figure 1B, both TSS and RS exhibited a progressive decline with extended fermentation duration. Following fermentation, the TSS in FPJ decreased from 20.31 °Brix to 16.46 °Brix (a 18.96% reduction), while RS levels dropped from 131.73 g/L to 94.13 g/L (a 28.56% decrease). This dual reduction is primarily attributed to the microbial utilization of reducing sugars by *Non-Saccharomyces cerevisiae* during fermentation, where sugars serve as the primary carbon source for yeast metabolism—generating ethanol, organic acids, and other byproducts—consequently driving the decline in TSS and available RS. During fermentation, lactic acid bacteria metabolize glucose and sucrose via the Embden–Meyerhof–Parnas pathway for homofermentative strains or the phosphoketolase pathway for heterofermentative strains [30]. This metabolic activity yields end products such as lactic acid (predominant in homofermentation), ethanol, and CO_2_ (characteristic of heterofermentation), thereby driving the depletion of soluble solids and reducing sugars. The observed reductions in TSS and RS are consistent with findings in fermented beverages, including wine, wheatgrass juice, kiwifruit juice, and pomegranate juice, where microbial sugar utilization is a universal driver of carbohydrate decline during fermentation [31,32,33].

### 3.3. Changes in TFC and TPC of FPJ During Different Fermentation Stages

As presented in Figure 1C, the TPC and total flavonoid content (TFC) in fermented prune juice increased by 20.28% and 28.95%, respectively, following fermentation. This augmentation is primarily attributed to microbial-mediated hydrolysis of covalent bonds linking polyphenols (including flavonoids) to structural macromolecules—such as pectin, cellulose, and proteins—in the plant cell wall. By disrupting these bonds, fermenting microorganisms (e.g., yeast and lactic acid bacteria, LAB) liberate bound phenolic compounds, converting them from conjugated or glycosylated forms into bioaccessible free phenolics [34].

Leonard et al. [35] demonstrated that synergistic interactions between yeast and LAB during co-fermentation enhance phenolic bioavailability by facilitating the breakdown of complex substrate matrices. Additionally, LAB-driven fermentation induces the depolymerization of macromolecular polyphenolic complexes (e.g., tannin–protein conjugates), a process that releases encapsulated phenolic moieties and increases the pool of extractable free phenolics [36].

### 3.4. Changes in DPPH and ABTS of FPJ During Different Fermentation Stages

As depicted in Figure 1D, both DPPH and ABTS radical scavenging capacities exhibited a consistent upward trend throughout fermentation, increasing by 76.16% and 57.25%, respectively, compared to pre-fermentation baselines. This indicates that fermentation significantly enhances (*p* < 0.05) the radical scavenging potential of FPJ, primarily attributed to two complementary mechanisms: (1) the microbial-mediated liberation of bound phenolic compounds (e.g., glycosylated flavonoids) from plant cell walls and (2) metabolic activities of lactic acid bacteria that generate bioactive byproducts with intrinsic antioxidant properties [37].

The observed augmentation in scavenging capacity aligns with the well-established correlation between polyphenol content and antioxidant activity. As reported by Lan et al. [38] in pomegranate wine fermentation, microbial transformations—such as the hydrolysis of glycosidic bonds and depolymerization of polyphenol–macromolecule complexes—increase the bioavailability of phenolic antioxidants, thereby enhancing radical scavenging efficiency. Additionally, the study links DPPH/ABTS scavenging capacity changes to fluctuations in the TPC and TFC (Figure 1C), consistent with the hypothesis that fermentation-induced increases in free polyphenols directly contribute to improved antioxidant capacity.

Malolactic fermentation, a key metabolic pathway in LAB-dominated systems, further elevates the levels of antioxidant-active compounds (e.g., hydroxycinnamic acids and anthocyanins) by converting less bioactive precursors into more potent scavengers [39].

### 3.5. Analysis of Volatile Compounds in Different Fermentation Stages of FPJ

**Figure 2:**
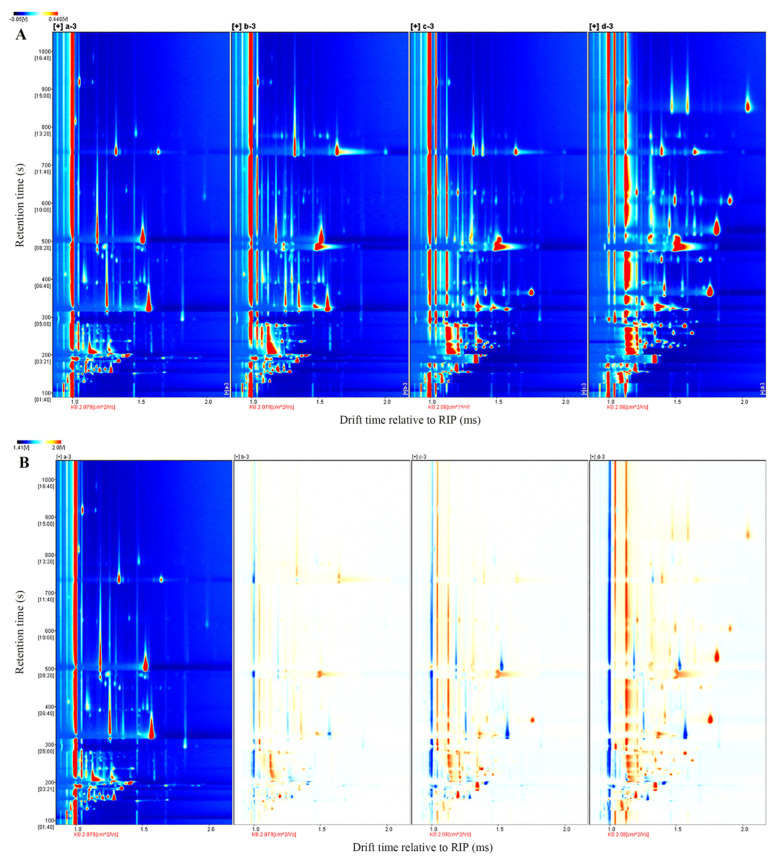
(**A**) Top view and (**B**) comparison of GC-IMS spectra of the FPJ at different fermentation stages. In the figure, a = PJ; b = YFPJ; c = LFPJ; and d = FPJ, The number 3 means three sets of parallel groups.

**Figure 3:**
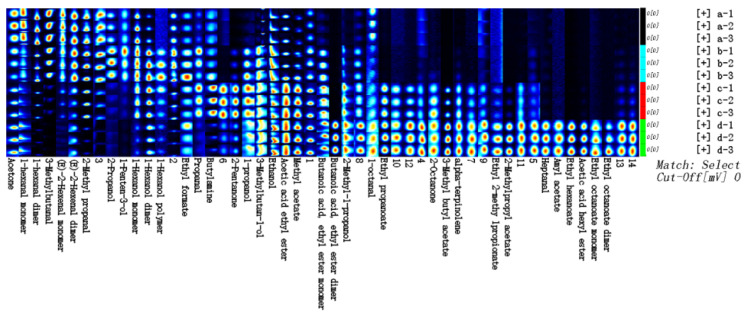
Fingerprinting plots of the volatile compounds in the FPJ at different fermentation stages. ^1^ In the figure, a = PJ; b = YFPJ; c = LFPJ; and d = FPJ. The numbers 1, 2, and 3 represent three parallel groups.

The two-dimensional GC-IMS spectra are presented in Figure 2A. Using the spectrum of unfermented PJ as the reference, spectral subtraction was performed on other samples to generate difference comparison plots (Figure 2B). In these plots, blue regions denote volatile compound concentrations lower than the reference, while red regions indicate higher concentrations. The x-axis represents the ion mobility time, and the y-axis corresponds to the gas chromatographic retention time. The results reveal that alterations in the ion peak count, color intensity (reflecting signal strength), and peak emergence timing directly reflect dynamic changes in the volatile compound profiles across different fermentation stages of FPJ. To characterize these stage-specific differences, the Gallery Plot plugin was employed to analyze signal intensities and generate volatile compound fingerprinting profiles, enabling straightforward visual comparison of compositional disparities between samples (Figure 3).

During the four fermentation stages of FPJ, 37 representative volatile compounds were identified, classified into 6 major functional groups: 14 esters, 8 alcohols, 7 aldehydes, 4 terpenes, 3 ketones, and 1 amine (Table 1). In PJ, acetone, hexanal, 3-methylbutyraldehyde, trans-2-hexenal, and isobutyraldehyde were the most concentrated, whereas their levels dropped significantly (*p* < 0.05) in FPJ. YFPJ exhibited elevated concentrations of ethanol, 1-penten-3-ol, isopropanol, hexanol, and ethyl formate. LFPJ was characterized by the highest levels of butylamine, methyl acetate, ethyl propionate, ethyl butyrate (M), ethyl acetate, isoamyl alcohol, propanal, 2-pentanone, and propanol. Notably, FPJ displayed peak abundances of ethyl butyrate, isobutanol, 2-octanone, isoamyl acetate, terpinolene, ethyl isobutyrate, iso-butyl acetate, hexyl acetate, heptanal, amyl acetate, ethyl hexanoate, and ethyl octanoate, compounds primarily associated with fruity, floral, and ester-like aromas.

**Figure 4:**
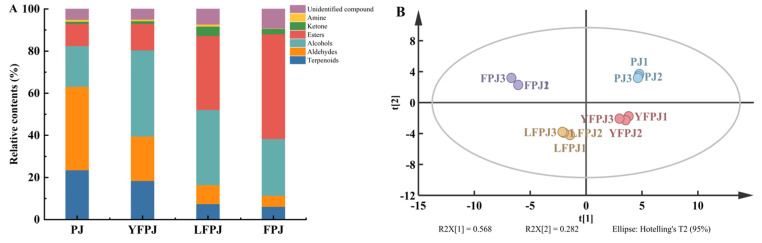

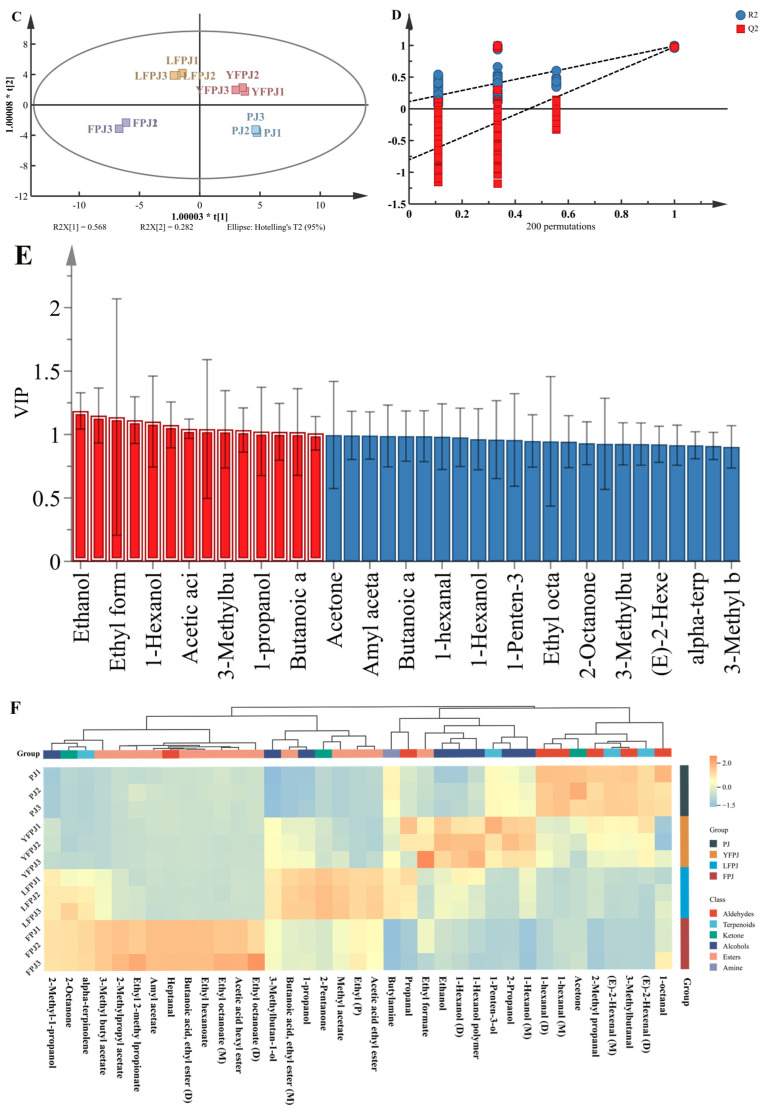
Analysis of volatile components of FPJ at different fermentation stages. (**A**) Variation in the relative content; (**B**) plot of PCA scores; (**C**) plot of OPLS-DA scores; (**D**) plot of validation of 200 substitutions; (**E**) VIP score plot; (**F**) clustering heatmap.

The relative abundances of volatile compounds across different fermentation stages are presented in Figure 4A. In PJ, aldehydes were the dominant class (39.63%), followed by terpenes (23.51%) and alcohols (19.32%). Among aldehydes, hexanal, 3-methylbutyraldehyde, and heptanal contributed distinct aromatic profiles to PJ, imparting fresh woody, chocolate-like, and crisp citrus notes, respectively. For YFPJ, alcohols became the primary volatile class (40.98%), followed by aldehydes (21.15%) and terpenes (18.46%), a shift likely driven by yeast-mediated ethanol production and aldehyde reduction. In LFPJ, alcohols remained dominant (36.02%), accompanied by a notable increase in esters (35.45%) and a decrease in aldehydes (9.05%). Alcohols play a pivotal role in shaping FPJ’s flavor architecture: propanol contributes fresh fruity sweetness, isopropanol adds subtle woody undertones, and isobutanol introduces whiskey-like complexities, collectively enhancing the beverage’s sensory depth. Isoamyl alcohol contributes to the floral and malt-like aromatic profiles of FPJ. Notably, esters constituted the most abundant volatile class in FPJ, accounting for 50.25% of the total profile, followed by alcohols (27.25%) and terpenes (6.25%). This substantial ester accumulation during fermentation is critical for shaping the beverage’s overall flavor complexity. Key ester compounds—including ethyl acetate, isoamyl acetate, iso-butyl acetate, methyl acetate, hexyl acetate, amyl acetate, ethyl hexanoate, ethyl octanoate (M), ethyl butyrate (M), and ethyl propionate—are primarily generated through enzyme-catalyzed esterification reactions between organic acids (e.g., acetic, hexanoic, and butyric acids) and alcohols (e.g., ethanol and isoamyl alcohol) [40]. These esters impart distinct fruity aromas to FPJ: ethyl acetate contributes a rich, grape-like fragrance with subtle floral undertones; ethyl hexanoate adds pronounced banana-like notes and tropical fruit nuances; and methyl acetate provides fresh, sweet fruity undertones, enhancing the beverage’s olfactory balance. Ketones and alcohols exhibited a biphasic trend of initial increase followed by decrease in relative abundance during fermentation. During this process, the biosynthesis of 2-octanone and 2-pentanone contributed distinct olfactory attributes to FPJ, imparting persistent woody, cheesy, and subtle fruity undertones. Meanwhile, aldehydes showed a consistent downward trend in relative abundance, primarily attributed to their inherent chemical instability in food matrices; aldehydes readily undergo reduction to alcohols or oxidation to organic acids under fermentation conditions [41]. High initial concentrations of aldehydes (e.g., hexanal and 3-methylbutyraldehyde) tend to generate undesirable pungent or harsh notes, whereas their gradual decline during fermentation mitigates off-flavor development. This aldehyde reduction significantly enhances the sensory acceptability of FPJ by promoting a more balanced and harmonious flavor profile, free from overwhelming sharpness associated with excessive aldehyde content. The decline in terpene relative abundance is likely attributed to biochemical transformations—including hydroxylation, acylation, isomerization, or oxidation—mediated by lactic acid bacteria, which convert terpenes into secondary metabolites (e.g., terpenols and esters). Sun et al. [42] demonstrated that LAB-dominated fermentation significantly reduces terpene levels in mixed-fruit juices, consistent with the observed trend in FPJ. Despite this reduction, terpenes generated during fermentation—such as monoterpenes and sesquiterpenes—contribute distinct aromatic attributes to FPJ, including fresh herbal notes, zesty lime nuances, subtle banana–cheese undertones, and resinous complexities.

Using the relative abundances of 37 volatile compounds identified across different fermentation stages, a PCA model was constructed for FPJ. As shown in the PCA score plot (Figure 4B), the four sample groups—PJ, YFPJ, LFPJ, and mixed-culture FPJ—exhibited distinct clustering, indicating clear differentiation in their volatile compound profiles. Additionally, data points from the same fermentation stage clustered tightly within the same principal component space, highlighting significant disparities in the composition of volatile compounds among the four groups.

Building on the PCA framework, an OPLS-DA model was constructed to facilitate robust sample discrimination. The model demonstrated excellent reliability, with fit parameters of R^2^X = 0.968, R^2^Y = 1, and Q^2^ = 0.976, indicating strong explanatory power for both the X (volatile compound matrix) and Y (sample group) datasets, as well as exceptional predictive ability. As illustrated in the OPLS-DA score plot (Figure 4C), the four sample groups—unfermented PJ, YFPJ, LFPJ, and mixed-culture FPJ—were distinctly separated along the discriminant components, highlighting clear compositional distinctions.

Model robustness was further validated via a 200-cycle permutation test (Figure 4D), where the intercept of the Q^2^ regression line on the y-axis was negative (below zero). This result confirms the absence of overfitting and underscores the model’s high predictive reliability, ensuring the validity of discriminant findings [43]. A total of 14 volatile compounds were identified as key discriminators across the four prune fermentation stages, with variable importance in projection (VIP) values ≥ 1 (Figure 4E). These included ethanol, hexanol (D), ethyl formate, 2-pentanone, hexanol (*p*), butylamine, ethyl acetate, octanal, isoamyl alcohol, methyl acetate, propanol, isopropanol, ethyl butyrate (M), and ethyl propionate, compounds whose concentration variations served as critical markers for differentiating volatile profiles among fermentation stages.

To characterize the dynamic changes in volatile profiles across different fermentation stages of FPJ, a heatmap was generated using the relative abundances of 37 identified volatile compounds (Figure 4F). Significant differences in volatile compound compositions were observed among sample groups. As fermentation advanced, characteristic volatile trends were classified into four major patterns: (1) upward trends (FPJ > LFPJ > YFPJ > PJ): compounds including terpinolene, isobutanol, isopropanol, isoamyl alcohol, hexyl acetate, and iso-butyl acetate exhibited a progressive increase in concentration, with the highest levels detected in mixed-culture fermented FPJ; (2) downward trends (FPJ < LFPJ < YFPJ < PJ): volatiles such as trans-2-hexenal, trans-3-hexenal, hexanal (D), isobutyraldehyde, 3-methylbutyraldehyde, and acetone displayed a gradual decline, with the sharpest reductions observed from unfermented PJ to FPJ; (3) the concentrations of 1-penten-3-ol, propanal, isopropanol, hexanol (P), hexanol (D), hexanol (M), propanol, ethyl butyrate (M), ethyl propionate, methyl acetate, 2-pentanone, ethanol, and ethyl formate exhibited a biphasic trend of initial increase followed by a decrease across fermentation stages; and (4) hexanal (M), isoamyl acetate, amyl acetate, ethyl hexanoate, ethyl octanoate (D), and ethyl octanoate (M) displayed a biphasic pattern of an initial decrease followed by an increase, reflecting dynamic metabolic transformations during fermentation.

### 3.6. Analysis of Organic Acids in FPJ During Fermentation

The concentrations of six major organic acids in FPJ at different fermentation stages are presented in Table 2. Lactic acid showed a significant increase during the lactic acid bacteria (LAB) fermentation phase, reaching its peak concentration in the late-stage LFPJ at 2415.17 ± 21.85 μg/mL. LAB metabolize sugars through fermentation pathways, producing significant amounts of organic acids, including lactic acid and acetic acid. This trend is consistent with the findings of Markakiou et al. [44], who reported increased levels of lactic and acetic acids in cheese fermented with *Lactiplantibacillus thermophilus* and *Lactococcus* spp., indicating a conserved microbial metabolic mechanism across diverse fermented food systems. The malic acid content reached its minimum (2.59 ± 0.01 μg/mL) at the end of FPJ fermentation, decreasing from an initial concentration of 1411.98 ± 3.58 μg/mL. Upon inoculation of YFPJ with *Lactiplantibacillus plantarum* to initiate lactic acid fermentation, malic acid in the fermentation broth was converted into lactic acid and carbon dioxide [38], leading to a significant decline (*p* < 0.05) in malic acid levels that exhibited an inverse correlation with lactic acid accumulation [45]. The tartaric acid content peaked in YFPJ at 4.71 ± 0.10 μg/mL, which was attributed to microbial degradation of tartrate salts during yeast fermentation, thereby increasing free tartaric acid concentrations [46]. Citric acid achieved its maximum concentration (51.49 ± 0.33 μg/mL) in FPJ, generated as an intermediate metabolite through glucose metabolism mediated by glycolysis and the tricarboxylic acid (TCA) cycle during fermentation [47]. D-glucuronic acid levels showed a continuous increase throughout fermentation, reaching 2965.31 ± 13.92 μg/mL in FPJ, as it was synthesized by yeast and lactic acid bacteria via glucose oxidation and glucuronic acid biosynthetic pathways [48]. The pantothenic acid content decreased markedly by the end of fermentation (28.88 ± 0.38 μg/mL compared to the initial 56.38 ± 4.32 μg/mL), primarily driven by its consumption during the rapid growth phase of yeast and lactic acid bacteria. Partial recovery occurred in later stages due to increased pantothenase activity mediating pantothenic acid catabolism [49].

**Figure 5:**
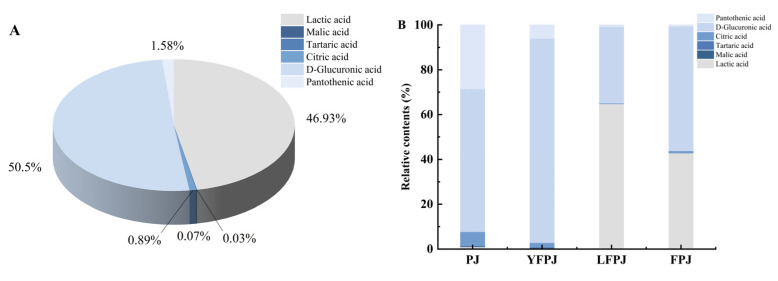

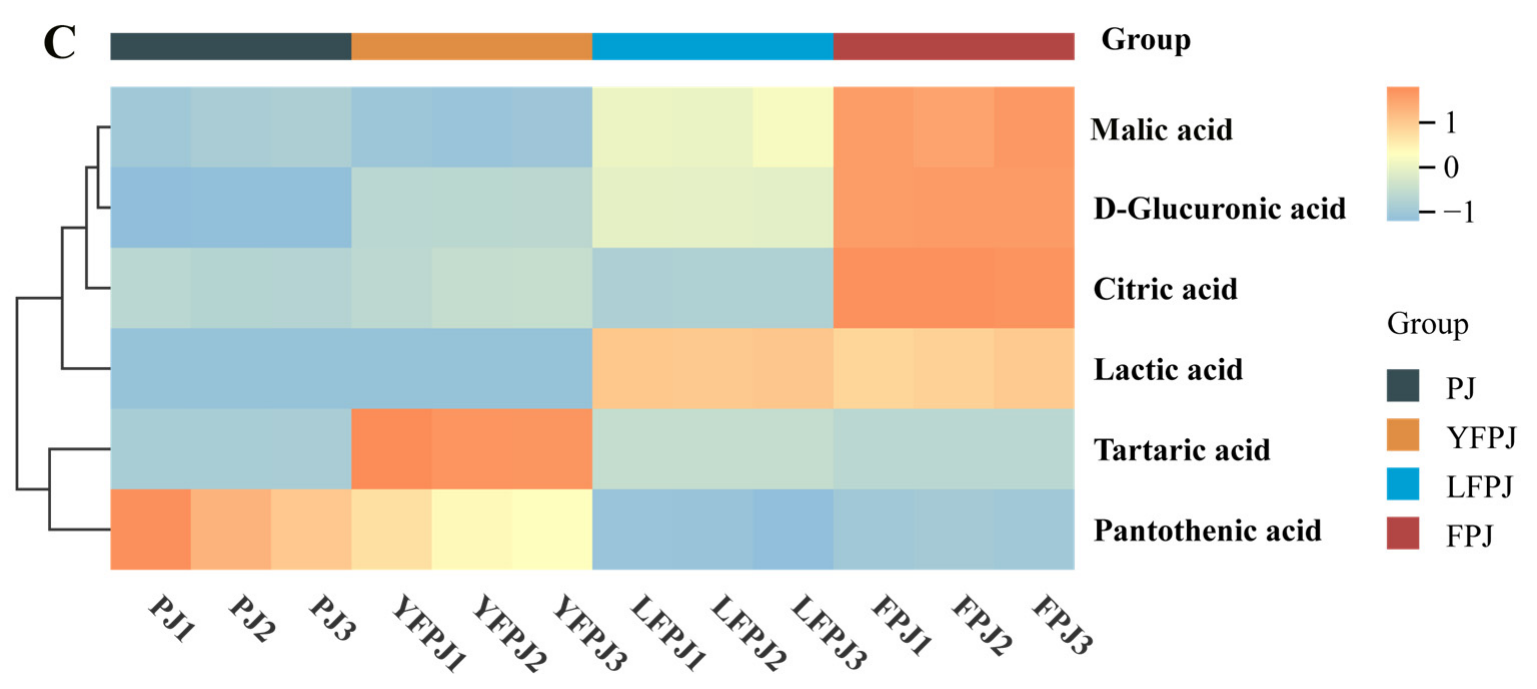
Analysis of organic acids of FPJ at different fermentation stages. (**A**) Total organic acid content distribution; (**B**) variation in the relative content; (**C**) clustering heatmap.

The distribution of six target organic acids at different fermentation stages is presented in Figure 5A. The dominant organic acids identified were D-glucuronic acid (50.50%), followed by lactic acid (46.93%) and pantothenic acid (1.58%), with tartaric acid exhibiting the lowest concentration (0.03%), correcting a potential term error, as malic acid was not listed among the analyzed acids in preceding sections. Figure 5B illustrates the quantitative profiles of organic acids across four fermentation phases. In the PJ phase, D-glucuronic acid constituted the primary component (63.76%), followed by pantothenic acid (28.56%) and citric acid (6.23%). The YFPJ phase showed a pronounced dominance of D-glucuronic acid (91.12%), followed by pantothenic acid (6.01%) and citric acid (1.96%), with tartaric acid detectable at minimal levels (0.06%). In contrast, lactic acid became the most abundant organic acid in the LFPJ phase (64.81%), with D-glucuronic acid accounting for 34.13% and tartaric acid showing the lowest content (0.03%). During the FPJ phase, D-glucuronic acid remained predominant at 55.64%, followed by lactic acid (42.81%), while the tartaric acid levels were negligible (0.01%).

The heatmap illustrating the concentrations of six organic acids across fermentation stages (Figure 5C) revealed significant compositional disparities among samples. Lactic acid, malic acid, and tartaric acid exhibited an initial increase followed by a gradual decline. Notably, the lactic acid levels remained consistently low and showed no significant differences during the PJ and YFPJ phases before surging to peak concentrations in the LFPJ phase. Although slightly reduced in the final FPJ phase, the lactic acid levels remained elevated. Conversely, malic acid started at a relatively high baseline in PJ, peaked in YFPJ, and then decreased to near-undetectable levels in both the LFPJ and FPJ phases. Tartaric acid reached its minimum in PJ and maximum in YFPJ, with concentrations significantly surpassing (*p* < 0.05) those of other phases. In contrast, citric acid and D-glucuronic acid displayed sustained increases. Citric acid remained stable in PJ and YFPJ but rose sharply in FPJ, achieving a 4.78-fold increase compared to LFPJ. D-Glucuronic acid accumulated steadily, culminating in FPJ at a concentration 23.56 times higher than in PJ. Pantothenic acid, conversely, showed a consistent downward trend, with no significant differences observed between the LFPJ and FPJ stages.

### 3.7. Analysis of Amino Acids in FPJ During Fermentation

As shown in Table 3, seven essential amino acids—valine (Val), threonine (Thr), leucine (Leu), isoleucine (Ile), lysine (Lys), phenylalanine (Phe), and histidine (His)—were detected at four fermentation stages of FPJ. These accounted for 4.70%, 4.17%, 3.65%, and 3.84% of the total free amino acids at each stage, respectively. Compared with PJ, the contents of leucine and isoleucine in YFPJ increased by 92.61% and 66.55%, respectively, which can be attributed to yeast fermentation promoting the synthesis of branched-chain amino acids [50]. Additionally, the Lys content in YFPJ rose by 155.53% relative to PJ, and in FPJ, it increased by 214.3% compared to the initial PJ stage. Ten non-essential amino acids—glycine (Gly), alanine (Ala), serine (Ser), proline (Pro), asparagine (Asn), aspartic acid (Asp), glutamine (Gln), glutamic acid (Glu), tyrosine (Tyr), and tryptophan (Trp)—were detected, accounting for 95.30%, 95.83%, 96.35%, and 96.16% of the total free amino acids in each sample, respectively. Functional amino acids exhibited significant increases (*p* < 0.05) during fermentation: the γ-aminobutyric acid (GABA) content increased by 27.05% in YFPJ compared to PJ and rose by 14.6% in FPJ relative to YFPJ. The proline (Pro) content increased by 9.35% in YFPJ compared to PJ, while FPJ contained 177.38 ± 2.64 μg/mL of Pro. The marked elevation in GABA and Pro levels suggests potential neuromodulatory, antioxidant, and anti-stress properties of FPJ [51,52]. Tyr and phenylalanine produced during fermentation may enhance FPJ’s antioxidant activity via mechanisms involving free radical scavenging and the inhibition of oxidative reactions [52].

**Figure 6:**
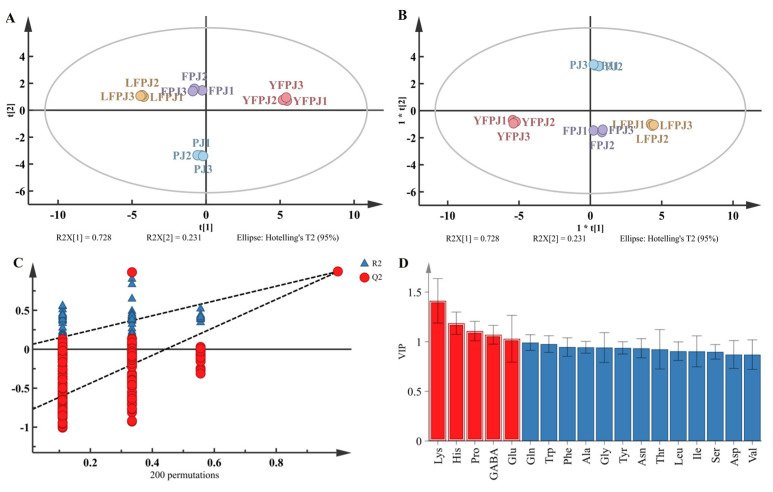
Analysis of amino acid of FPJ at different fermentation stages. (**A**) Plot of PCA scores; (**B**) plot of OPLS-DA scores; (**C**) plot of validation of 200 substitutions; (**D**) VIP score plot.

Using the amino acid profiles obtained during the fermentation of FPJ, a PCA model was constructed with 18 free amino acids identified at various fermentation stages. As illustrated in the PCA score plot (Figure 6A), the PJ, YFPJ, LFPJ, and FPJ groups were distinctly separated. Significantly, data points from juices at the same fermentation stage were closely clustered in specific regions, suggesting significant differences in the free amino acid profiles among the four groups.

Building on the PCA results, an OPLS-DA model was developed to improve inter-group discrimination (*R*^2^X = 0.996; *R*^2^Y = 0.998; *Q*^2^ = 0.997), as shown in Figure 6B, which clearly separated the four fermentation groups. A 200-cycle permutation test (Figure 6C) validated the model’s reliability: the Y-intercept of the *Q*^2^ regression line was below zero, confirming no overfitting and robust predictive performance [43]. Five amino acids with variable importance in projection (VIP ≥ 1)—Lys, His, Pro, GABA, and Glu—were identified as critical discriminators across fermentation stages (Figure 6D). Specifically, the dynamic profiles of these amino acids served as biochemical markers to distinguish FPJ at distinct fermentation phases.

**Figure 7:**
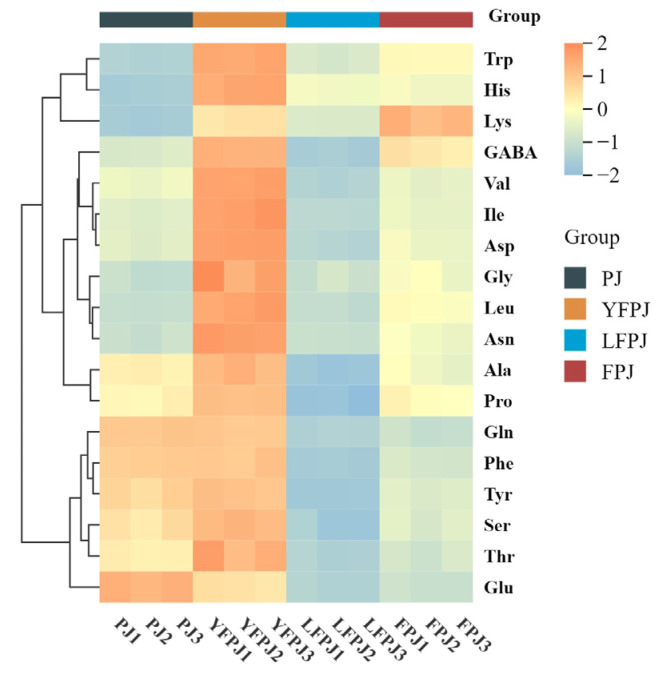
Amino acid clustering heatmap at different fermentation stages of FPJ.

To characterize the variations in amino acid profiles of FPJ across different fermentation stages, a heatmap was generated using the contents of 18 detected amino acids, as shown in Figure 7. The dynamic changes of amino acids during the fermentation process were classified into four distinct patterns: (1) GABA, Lys, His, Asn, Trp, Asp, and Arg exhibited a consistently increasing trend (FPJ > PJ); (2) Ala, Ser, Pro, Glu, Tyr, and Gln displayed a consistently decreasing trend (FPJ < PJ); (3) Phe, Thr, Val, Ile, Leu, along with GABA, Lys, His, Asn, Asp, Trp, Ala, Ser, and Pro, followed a triphasic pattern: an initial increase, a subsequent decrease, and a final renewal of increase; and (4) Glu showed a biphasic profile with an initial decrease followed by a gradual increase.

## 4. Conclusions

This study employed prunes as the raw material to prepare FPJ through inoculation with yeast and lactic acid bacteria. The physicochemical properties, antioxidant capacity, and volatile compounds were analyzed for four sample groups: PJ, YFPJ, LFPJ, and FPJ. The fermentation process reduced FPJ’s pH, TSS, and RS content while significantly increasing (*p* < 0.05) the TPC and TFC. Compared with PJ, FPJ exhibited significant increases (*p* < 0.05) in the TPC, TFC, DPPH radical scavenging capacity, and ABTS radical scavenging capacity, with increases of 20.28%, 28.95%, 76.16%, and 57.25%, respectively. A total of 37 representative volatile compounds were identified across the four sample groups, comprising 14 esters, 8 alcohols, 7 aldehydes, 4 terpenes, 3 ketones, and 1 amine. Multivariate statistical analysis revealed significant differences in flavor profiles among fermented prune juice samples at distinct fermentation stages. Key discriminative volatile compounds contributing to group discrimination included ethanol, hexanol (D), ethyl formate, 2-pentanone, hexanol (P), butylamine, ethyl acetate, octanal, isoamyl alcohol, methyl acetate, propanol, isopropanol, ethyl butyrate (M), and ethyl propionate, with their concentration variations driving the differentiation. Significant levels of organic acids and free amino acids were detected in the four samples from different fermentation stages. Notably, the concentrations of lactic acid, citric acid, and D-glucuronic acid increased significantly (*p* < 0.05), reaching 2281.406 ± 87.498 μg/mL, 51.494 ± 0.329 μg/mL, and 2965.305 ± 13.923 μg/mL, respectively. Among the free amino acids in FPJ, Asn had the highest concentration (2305.849 ± 65.014 μg/mL), followed by Gln (265.046 ± 7.434 μg/mL) and Pro (177.377 ± 2.642 μg/mL). Fermentation with yeast and lactic acid bacteria altered the sugar–acid ratio of FPJ, increased the TPC and TFC, enhanced the antioxidant capacity, optimized the characteristic aroma and flavor profiles, enriched nutritional components, and ultimately improved both the sensory quality and functional properties of FPJ. FPJ demonstrates substantial nutritional value and functional attributes, positioning it as a promising candidate for functional food development. Future research should prioritize elucidating the interactions between microbial community dynamics and metabolite profiles during fermentation, thereby providing valuable insights for process optimization and product quality enhancement.

## Figures and Tables

**Table 1:** Changes in volatile compounds and their relative contents in the FPJ at different fermentation stages.

Volatile Compound	Ri	Rt	Dt	Odor Characteristics	Relative Contents (%)			
					PJ	YFPJ	LFPJ	FPJ
Terpenoids								
(E)-2-Hexenal (D)	1228.8	504.467	1.52307	Banana, fruit fragrance, and cheese	14.87 ± 0.06 ^a^	12.91 ± 0.01 ^b^	5.10 ± 0.01 ^c^	4.38 ± 0.00 ^c^
(E)-2-Hexenal (M)	1229.6	505.801	1.18269	Green leaves, botany aroma, and fat	8.19 ± 0.04 ^a^	5.00 ± 0.01 ^b^	1.26 ± 0.00 ^c^	0.57 ± 0.00 ^d^
alpha-terpinolene	1294.7	627.58	1.21139	Lemon and lime flavor	0.15 ± 0.16 ^c^	0.22 ± 0.00 ^c^	1.04 ± 0.00 ^b^	1.31 ± 0.00 ^a^
1-Penten-3-ol	1169.1	412.584	0.94462	Green radish, resin, and fruit	0.29 ± 0.00 ^b^	0.40 ± 0.00 ^a^	0.10 ± 0.00 ^c^	0.04 ± 0.00 ^d^
Aldehydes								
1-hexanal (D)	1098.3	323.086	1.56687	Grassy and fruity	21.83 ± 0.95 ^a^	10.46 ± 0.02 ^b^	3.89 ± 0.00 ^c^	1.52 ± 0.00 ^d^
1-hexanal (M)	1098.3	323.086	1.25783	Grassy and fruity	6.89 ± 0.03 ^a^	3.38 ± 0.00 ^b^	1.95 ± 0.00 ^d^	2.24 ± 0.00 ^c^
1-octanal	1294.5	627.164	1.40345	Orange and tangerine	0.29 ± 0.01 ^a^	0.21 ± 0.00 ^b^	0.23 ± 0.00 ^b^	0.25 ± 0.00 ^b^
2-Methyl propanal	834.6	158.601	1.28919	Green malt	4.57 ± 0.20 ^a^	3.21 ± 0.01 ^b^	1.71 ± 0.00 ^c^	0.35 ± 0.00 ^d^
3-Methylbutanal	928.2	200.358	1.41049	Cocoa aroma	5.59 ± 0.24 ^a^	3.20 ± 0.01 ^b^	0.59 ± 0.00 ^c^	0.10 ± 0.00 ^c^
Heptanal	1195	451.023	1.68912	Citrusy	0.12 ± 0.01 ^b^	0.11 ± 0.00 ^b^	0.09 ± 0.00 ^b^	0.71 ± 0.00 ^a^
Propanal	793.3	143.092	1.04329	Wine and whiskey	0.34 ± 0.02 ^b^	0.56 ± 0.00 ^a^	0.60 ± 0.00 ^a^	0.14 ± 0.00 ^b^
Alcohols								
1-Hexanol (D)	1372.4	737.624	1.64338	Fruity and alcoholic	1.56 ± 0.07 ^c^	5.42 ± 0.01 ^a^	3.72 ± 0.01 ^b^	2.09 ± 0.00 ^c^
1-Hexanol (M)	1371.8	736.612	1.32696	Fruity and alcoholic	4.16 ± 0.42 ^b^	6.10 ± 0.00 ^a^	3.95 ± 0.00 ^b^	1.18 ± 0.00 ^c^
1-Hexanol (P)	1370.4	734.587	2.00646	Fruity and alcoholic	0.17 ± 0.02 ^b^	0.36 ± 0.00 ^a^	0.22 ± 0.00 ^b^	0.13 ± 0.00 ^c^
1-propanol	1051.4	280.434	1.25231	Fruity aroma of bubble gum	0.22 ± 0.02 ^c^	0.77 ± 0.00 ^b^	1.28 ± 0.00 ^a^	0.68 ± 0.00 ^b^
2-Methyl-1-propanol	1102	327.211	1.37593	Whisky	0.51 ± 0.05 ^d^	1.50 ± 0.00 ^c^	3.55 ± 0.00 ^b^	3.81 ± 0.00 ^a^
2-Propanol	919.5	196.093	1.21602	Alcohol and wood	0.45 ± 0.04 ^b^	0.69 ± 0.00 ^a^	0.12 ± 0.00 ^c^	0.05 ± 0.00 ^c^
3-Methylbutan-1-ol	1216.2	483.758	1.48789	Potpourri, malt, and empyreumatic	2.14 ± 0.02 ^d^	8.27 ± 0.00 ^b^	9.33 ± 0.00 ^a^	7.72 ± 0.00 ^c^
Ethanol	951.3	212.289	1.13151	Alcoholic	10.11 ± 0.01 ^d^	17.93 ± 0.01 ^a^	13.84 ± 0.00 ^b^	11.79 ± 0.00 ^c^
Amine								
Butylamine	924.2	198.372	1.37778	Ammoniacal fishy smell	0.92 ± 0.09 ^a^	0.77 ± 0.00 ^b^	1.02 ± 0.00 ^a^	0.18 ± 0.00 ^c^
Esters								
2-Methylpropyl acetate	1026.6	260.21	1.62496	Sweet fruit and banana	0.08 ± 0.01 ^c^	0.06 ± 0.00 ^c^	0.17 ± 0.00 ^b^	0.82 ± 0.00 ^a^
3-Methyl butyl acetate	1134.5	366.059	1.75684	Sweet fruit and banana	0.41 ± 0.04 ^c^	0.33 ± 0.00 ^c^	4.70 ± 0.00 ^b^	9.48 ± 0.00 ^a^
Acetic acid ethyl ester	900.2	186.837	1.34433	Grape flavor and sweet	4.74 ± 0.00 ^c^	4.56 ± 0.01 ^c^	15.90 ± 0.01 ^a^	11.90 ± 0.00 ^b^
Acetic acid hexyl ester	1283.6	605.021	1.9045	Apple and pear	0.25 ± 0.00 ^b^	0.23 ± 0.00 ^b^	0.27 ± 0.00 ^b^	1.95 ± 0.00 ^a^
Amyl acetate	1195	451.023	1.76143	Banana and sweet	0.05 ± 0.00 ^b^	0.05 ± 0.00 ^b^	0.05 ± 0.00 ^b^	0.21 ± 0.00 ^a^
Butanoic acid, ethyl ester (D)	1047	276.714	1.57369	Apple smell and sweet	0.20 ± 0.00 ^b^	0.21 ± 0.00 ^b^	0.18 ± 0.00 ^b^	2.09 ± 0.00 ^a^
Butanoic acid, ethyl ester (M)	1049.4	278.702	1.19216	Apple smell and sweet	0.39 ± 0.00 ^d^	1.23 ± 0.00 ^b^	1.83 ± 0.00 ^a^	1.09 ± 0.00 ^c^
Ethyl 2-methy lpropionate	976.6	226.106	1.57167	Rum	0.04 ± 0.00 ^b^	0.04 ± 0.00 ^b^	0.04 ± 0.00 ^b^	0.08 ± 0.00 ^a^
Ethyl formate	814	150.648	1.07417	Fruity and winy	0.47 ± 0.00 ^b^	0.77 ± 0.00 ^a^	0.49 ± 0.00 ^b^	0.54 ± 0.00 ^b^
Ethyl hexanoate	1242.6	528.039	1.80647	Banana and pineapple	0.74 ± 0.00 ^b^	0.54 ± 0.00 ^c^	0.41 ± 0.00 ^c^	10.54 ± 0.00 ^a^
Ethyl octanoate (D)	1443.7	855.07	2.03485	Waxy taste and fruity	0.40 ± 0.00 ^b^	0.33 ± 0.00 ^b^	0.32 ± 0.00 ^b^	2.46 ± 0.01 ^a^
Ethyl octanoate (M)	1441.4	851.02	1.473	Waxy taste and fruity	0.48 ± 0.00 ^b^	0.37 ± 0.00 ^c^	0.33 ± 0.00 ^c^	1.86 ± 0.00 ^a^
Ethyl propanoate	968.4	221.547	1.46176	Sweet and fruity	0.07 ± 0.00 ^c^	0.07 ± 0.00 ^c^	0.99 ± 0.00 ^a^	0.77 ± 0.00 ^b^
Methyl acetate	850.3	164.964	1.20208	Fruity and winy	2.25 ± 0.00 ^d^	3.69 ± 0.01 ^c^	9.79 ± 0.00 ^a^	6.20 ± 0.00 ^b^
Ketone								
2-Octanone	1294.4	626.936	1.34411	Woody fragrance, cheese, and yeast	0.06 ± 0.00 ^b^	0.07 ± 0.00 ^b^	0.30 ± 0.00 ^a^	0.33 ± 0.00 ^a^
2-Pentanone	996	237.343	1.37741	Fruit and pungent	0.19 ± 0.00 ^c^	0.71 ± 0.00 ^b^	3.06 ± 0.00 ^a^	0.83 ± 0.00 ^b^
Acetone	831.5	157.408	1.12158	Apple and pear	0.79 ± 0.00 ^a^	0.36 ± 0.00 ^b^	0.28 ± 0.00 ^b^	0.29 ± 0.00 ^b^

Letters in superscript in the table data indicate significant differences (*p* < 0.05). Flavor compounds labeled with M, D, and P represent monomer, dimer, and polymer forms, respectively. In the samples, PJ, YFPJ, LFPJ, and FPJ denote prune juice, yeast-fermented prune juice, lactic acid bacteria-fermented prune juice, and fermented prune juice, respectively. Odor characteristics were retrieved from the Flavor Ingredient Library database using the CAS numbers of the substances.

**Table 2:** Changes in organic acid types and their contents in the FPJ at different fermentation stages.

Organic Acids	Contents (μg/mL)			
	PJ	YFPJ	LFPJ	FPJ
Lactic acid	1.96 ± 0.20 ^c^	1.59 ± 0.19 ^c^	2415.17 ± 21.85 ^a^	2281.41 ± 87.50 ^b^
Malic acid	1411.98 ± 3.58 ^b^	2571.48 ± 13.39 ^a^	4.04 ± 0.04 ^c^	2.59 ± 0.01 ^d^
Tartaric acid	0.33 ± 0.02 ^d^	4.71 ± 0.10 ^a^	0.97 ± 0.01 ^b^	0.72 ± 0.00 ^c^
Citric acid	12.31 ± 0.81 ^c^	14.94 ± 1.15 ^b^	10.78 ± 0.29 ^d^	51.49 ± 0.33 ^a^
D-Glucuronic acid	125.86 ± 8.05 ^d^	693.94 ± 10.48 ^c^	1272.03 ± 18.57 ^b^	2965.31 ± 13.92 ^a^
Pantothenic acid	56.38 ± 4.32 ^a^	45.77 ± 2.52 ^b^	27.02 ± 0.58 ^d^	28.88 ± 0.38 ^c^

Letters in superscript in the table data indicate significant differences (*p* < 0.05).

**Table 3:** Changes in amino acid species and their contents in the FPJ at different fermentation stages.

Free Amino Acids	Contents (μg/mL)			
	PJ	YFPJ	LFPJ	FPJ
Gly	0.46 ± 0.03 ^d^	1.24 ± 0.10 ^a^	0.51 ± 0.05 ^c^	0.72 ± 0.06 ^b^
Ala	28.50 ± 0.20 ^b^	31.55 ± 0.41 ^a^	22.09 ± 0.18 ^d^	26.92 ± 0.78 ^c^
GABA	49.71 ± 0.41 ^c^	63.14 ± 0.20 ^a^	43.72 ± 0.39 ^d^	57.01 ± 0.95 ^b^
Ser	24.16 ± 0.49 ^ab^	26.43 ± 0.22 ^a^	17.33 ± 0.64 ^c^	20.65 ± 0.42 ^b^
Pro	179.23 ± 1.50 ^b^	195.97 ± 0.53 ^a^	140.98 ± 1.34 ^c^	177.38 ± 2.64 ^b^
Val	21.83 ± 0.36 ^b^	31.89 ± 0.23 ^a^	16.39 ± 0.17 ^c^	21.29 ± 0.44 ^b^
Thr	26.80 ± 0.20 ^b^	31.16 ± 1.08 ^a^	20.22 ± 0.32 ^c^	22.66 ± 0.49 ^c^
Ile	8.34 ± 0.09 ^b^	13.89 ± 0.27 ^a^	6.81 ± 0.07 ^c^	8.78 ± 0.18 ^b^
Leu	5.04 ± 0.02 ^c^	9.71 ± 0.23 ^a^	4.91 ± 0.11 ^c^	6.91 ± 0.15 ^b^
Asn	1982.76 ± 41.47 ^c^	3113.73 ± 34.75 ^a^	1973.04 ± 12.49 ^c^	2305.85 ± 65.01 ^b^
Asp	152.23 ± 1.88 ^b^	206.68 ± 0.95 ^a^	133.36 ± 2.01 ^c^	158.20 ± 3.33 ^b^
Gln	395.81 ± 3.27 ^a^	393.55 ± 4.30 ^a^	236.73 ± 2.89 ^c^	265.05 ± 7.43 ^b^
Lys	0.80 ± 0.02 ^d^	2.03 ± 0.04 ^b^	1.36 ± 0.01 ^c^	2.50 ± 0.09 ^a^
Glu	110.50 ± 1.43 ^a^	95.02 ± 1.50 ^b^	60.40 ± 1.16 ^d^	68.34 ± 1.14 ^c^
His	3.48 ± 0.15 ^c^	11.22 ± 0.25 ^a^	6.85 ± 0.09 ^b^	6.82 ± 0.20 ^b^
Phe	77.25 ± 0.76 ^a^	78.92 ± 1.28 ^a^	40.82 ± 0.46 ^c^	53.20 ± 1.08 ^b^
Tyr	36.80 ± 0.86 ^a^	38.93 ± 0.54 ^a^	21.16 ± 0.11 ^c^	28.36 ± 0.51 ^b^
Trp	2.30 ± 0.03 ^d^	6.31 ± 0.08 ^a^	3.24 ± 0.07 ^c^	4.35 ± 0.01 ^b^

Letters in superscript in the table data indicate significant differences (*p* < 0.05).

## Data Availability

The original contributions presented in this study are included in the article. Further inquiries can be directed to the corresponding author.

## Abbreviations

The following abbreviations are used in this manuscript:

PJ	Prune juice
YFPJ	Yeast fermentation prune juice
LFPJ	Lactic acid fermentation prune juice
FPJ	Fermented prune juice
ABTS	2,2’-azinobis-(3-ethylbenzothiazoline-6-sulfonate acid)
DPPH	1,1-diphenyl-2-picrylhydrazyl
GC-IMS	Gas chromatography–ion mobility spectrometry
LC-MS	Liquid chromatography–mass spectrometry
PCA	Principal component analysis
OPLS-DA	Orthogonal partial least squares discriminant analysis
TA	Titratable acidity
TSS	Total soluble solids
RS	Reducing sugars
TPC	Total phenolic content
TFC	Total flavonoid content
VIP	Variable importance in projection
M	Monomer
D	Dimer
P	Polymer
